# Book Reviews

**Published:** 1831-12

**Authors:** 


### BIBLIOGRAPHICAL NOTICES.

*A Practical Treatise on Injuries of the Head.*—12mo. pp. 121. Fannin and Co., Dublin.

ANONYMOUS works are usually consulted with some degree of hesitation, and in many, or perhaps most cases, they do not merit a high degree of confidence. The present practical treatise forms an exception to the general rule, for it contains a most excellent digest of the best opinions that have been given upon the important subject of diseases of the head. If it should pass to a second edition, which we think is more than probable, we would advise the author to place his name in the title-page. If he be known as a veteran in surgery, his work will, of course, be more eagerly sought after; and, if he be a tyro in his art, he will gain some steps in reputation by the judicious arrangement of this little work, and the correct practical information he has collected for the service of his brother students.

It is stated in the preface, that the volume has been principally compiled from the works of the most eminent writers on injuries of the head. "Thanks to the proverbial pugnacity of our warm-hearted and hot-headed countrymen, the hospitals of Dublin present ample opportunities for studying the subject in all its varieties: these opportunities, it will be found, have not been totally neglected."

This treatise is particularly well adapted to the capacity of students: it combines the latest improvements, with the *present practice of hospital surgeons* in injuries of the head, and every part of the subject is clearly yet concisely explained. At the end of each chapter, its substance is given in the form of aphorisms. The idea of this plan was taken from DEASE's Aphorisms, and from Mr. COLLES's valuable little work on Injuries of the Head. The student will find these brief recapitulations of great service in fixing upon his memory the most important practical points. In fine, the author has collected, from the best authorities, and in a very cheap and portable form, all the information which the student or practitioner can require upon the subject of injuries of the head.

---

*Elements of Botany: consisting of the Anatomy and Physiology of Plants; an Explanation of the Classification of Plants by Linnæus; an Arrangement of Medicinal Plants, and a Glossary of Botanical Terms. Second Edition, with Plates.* By JOHN STEGGALL, M.D. &c.—12mo. pp. 155. Highley, London.

COMPARED with contemporary publications of similar size, and ushered into the world with similar pretensions, Dr. STEGGALL's work is by far the best that we as yet have seen: it is, indeed, the only popular Manual which can be at all conscientiously recommended to students, as it is the only one that contains (slight as the sketch necessarily is) any thing which can be considered even a glimpse, not to talk of a view, of modern Botany; i. e. which is written with a knowledge of the numerous discoveries lately made in this branch of natural science.

This alone would ensure from us a favorable notice, and, were the volume incapable of improvement, we should rest content with affording it a general commendation; but the more we like a book, the more intolerant do we become of its defects, and what would pass unnoticed in a worse cannot pass uncensured in a better.

Dr. Steggall, in this work, an avowed compilation from the writings of De Candolle, Mirbel, Gærtner, and Richard, has followed closely the authorities taken for his guides, and in so doing he has done well; but he would have done much better had he collated their opinions, and corrected their views by still later experiments and recent observations. Had Dr. Steggall duly estimated the experiments of Schultz and others, who have absolutely shewn the fluids moving *in the vessels* of plants, and, by sulphate of iron, have discoloured the fluids impregnated with ferro-cyanate of potash contained in the vessels of living vegetables, he would have qualified considerably his quotations from Keyser, and therefore the sooner such dogmas as the following are altered the better:

"The *passages between the cells* surrounding the vessels are subservient to the ascent of the sap from the root." (P. 7.)

"The *vessels* are described by some botanists as subservient to the passage of the sap: this opinion, however, is feebly supported, for they are *not found to contain any fluid*," &c. (P. 10.)

"The duramen and alburnum are the principal, and nearly the sole support of the tree; they serve also as a medium for the fluids to pass up from the roots, through the *intercellular passages* existing in them." (P. 31.)

Indeed, had Dr. S. reflected on what he was writing, the following irreconcileable statement would never have appeared in the same book, and especially in the same edition with the foregoing: "The fluids absorbed by the roots or leaves are conveyed to the entire vegetable by the *large vessels* of the wood, and especially by the soft layers of the alburnum." (P. 86.)

The physiology of the motion of the sap is also very far less satisfactory than it need have been left.

"Roots are distinguished from stems by many marked characters: there *is a total absence of spiral vessels* and stomata. The internal structure of the roots of endogenous plants differs but little from that of their stems; for they are made up of dotted and radiated vessels, intermixed with cellular tissue. The difference between the two in exogenous plants is much greater: in the roots there is *no medullary canal or pith.*"

This is another proof of the extent to which dicta are copied, and opinions adopted, without examination: for Link and Treviranus have long shewn the existence of spiral vessels in the roots of several plants; Amici has discovered them in several, and we have ourselves seen them also; e. g. they are very evident in the roots of Agapanthus umbellatus: and as to the presence of pith in roots, any one who will take the trouble to slit a young horse-chesnut, as described by Richard, will at once be convinced of its abundance.

We pass over various other proofs of haste or negligence, recommending them to the author's revisal; for, although in some measure excusable in a first, they ought to have been corrected in a second edition: e. g. the obsolete doctrine concerning the calyx being "an expansion or elongation of the bark;" the corolla "a continuation of the ligneous substance situated under the bark," &c. But we must enter our more formal protest against the doctrine laid down, page 60, that "there are no seeds which are naked, that is, not covered by a pericarp," (p. 60;) for, although the gymnospermous plants of Linnæus have pericarps, no one has ever shewn a seed-vessel to exist in the Corriferæ; and how would a student, with reference to the above, be astonished to meet with the following definition of the first order of the fourteenth class Didynamia: "Order 1. *Gymnospermia* has its *naked seeds*, which are usually situated at the bottom of the calyx." (P. 93.)

The definition of the third order of the nineteenth class is unsatisfactory. "Order 3. *Polygamia frustanea* has the florets of the disk perfect, and those of the circumference barren." Now, what are the author's own definition of the terms employed? vide Glossary, p. 108: "Barren flowers are flowers with *stamens* only, and which produce no fruit." But are the florets in the circumference in the order in question furnished with stamens, and with stamens only? No; they have not any stamens at all; but they have pistils, and they bear no seeds, not on account of the absence of pistils, but on account of their imperfect organization; and hence, indeed, is it that the polygamy is frustraneous, or in vain.

There are several errors likewise in the Glossary; but we have probably said enough to excite the author's attention, and more than enough to arouse the student's curiosity, to whom we recommend a perusal of this little book for, notwithstanding its several faults, we like it still.

---

*The Elements of Chemistry, familiarly explained, and practically illustrated. Part the First. Attraction, Heat, Light, Electricity.*—8vo. pp. 318. John Murray, London.

Notwithstanding the almost unlimited delight with which an intelligent student pursues the science of chemistry, when he has once acquired a thorough knowledge of its fundamental principles, he will find the commencement of his progress dull and almost disheartening, unless he make a happy selection of some *elementary* work for his guide, in which the first steps of chemical philosophy are explained in a clear and familiar manner. Knowledge can be obtained only by degrees, and he who ventures at once into the utmost depths of an intricate and difficult science, not unfrequently abandons his task in despair, and attributes his failure to his own want of ability, when the injudicious mode in which the study has been commenced is alone in fault.

The little volume before us, which is but the first of others to follow upon the "Elements of Chemistry," is much better adapted than any other with which we are acquainted to carry the student on in an easy and instructive manner. The great superiority which it possesses over other similar works that have previously been published, consists in the numerous cuts which it contains. Verbal descriptions alone, however clear may be the diction, must often fail in impressing upon the mind of a beginner a perfect comprehension of many of those important facts upon which the whole doctrines of chemistry rest. In this single volume we have a hundred engravings illustrative of the text, and upon the same page with the verbal description of the subject: the great assistance the student will hence derive can only be fully appreciated by comparing the comparatively little progress that can be made by the study of a work without engravings, with that which will be effected by the study of another with graphic illustrations.

The present volume includes an outline of the subjects of Attraction, Heat, Light, and Electricity. The author has judiciously avoided all prolixity of language and the use of the less intelligible technical terms. Other volumes will be subsequently published, containing the more practical and ordinary details of the chemical laboratory, and concluding with a general history of the rise and progress of the science.

